# A Spontaneous Groin Collection Masquerading as an Irreducible Inguinoscrotal Hernia

**DOI:** 10.7759/cureus.27563

**Published:** 2022-08-01

**Authors:** Sadiq Mawji, Elizabeth Peterknecht, Shafquat Zaman, Ali Yasen Y Mohamedahmed, Dham Mobarak

**Affiliations:** 1 General Surgery, Sandwell and West Birmingham NHS Trust, Birmingham, GBR

**Keywords:** irreducible hernia, laparoscopic surgery, spontaneous rupture, inferior epigastric vessels, inguinal hernia

## Abstract

This case report details a clinically rare presentation in which a middle-aged man was diagnosed clinically with a large irreducible inguinoscrotal hernia. However, intraoperatively, a large volume of old blood/clots was seen and aspirated, without a definite hernia being identified. Inguinal hernias remain a clinical diagnosis, and imaging is used only in equivocal cases. Owing to the number of differential diagnoses associated with groin swelling, careful clinical assessment is critical in differentiating between the various causes. Rupture of the inferior epigastric vessels was suspected, and although rare, it should be considered as a differential diagnosis as this may alter ongoing management.

## Introduction

The inferior epigastric artery originates from the external iliac artery just above the inguinal ligament. It ascends superiorly and medially toward the umbilicus and supplies the muscles of the anterior abdominal wall. It forms an anastomosis with the superior epigastric artery and is accompanied by the inferior epigastric vein along its course [1].

Owing to this anatomical location, these vessels can be injured and can lead to life-threatening haemorrhage. Iatrogenic injury from the insertion of lateral laparoscopic trocars has been reported in up to 2% of operative laparoscopies [1,2]. They can also rupture through blunt or penetrating abdominal trauma, or even through persistent heavy coughing, sneezing, or rapid abdominal movements [3,4].

Spontaneous inferior epigastric vessel rupture is associated with sudden-onset acute abdominal pain, swelling, and rectus sheath haematoma [5]. Treatment is largely conservative and mainly consists of a period of observation, analgesia, and, in selected cases, antibiotics. Where applicable, anti-coagulants are often suspended.

Spontaneous rupture of the vessels is a rarely reported phenomenon [3,4] with limited case reports available in the literature. Of these, few describe the presenting complaint as a swelling in the groin [6,7]. Two case reports in the literature outline patients presenting with abdominal swelling, haemodynamic instability, and active haemorrhage [5-8] who were managed surgically.

We report on a rare clinical presentation in which a large collection of old blood was found masquerading as a large inguinoscrotal hernia. The source of the bleeding was suspected to have arisen from the inferior epigastric vessels.

## Case presentation

A 66-year-old Asian man presented to the surgical outpatient clinic with a 12-month history of worsening left-sided groin pain and swelling. His symptoms had recently begun to worsen with increasing discomfort, but despite this, he continued to work full time in a manual job. He denied any nausea or vomiting and there were no bowel or urinary symptoms. Furthermore, he was systemically well with no chest pain, dyspnoea, or dizziness.

The patient’s medical history included type 2 diabetes and hypertension. He had no previous significant surgical history, and his daily medication included metformin, amlodipine, and ramipril. He had no known drug allergies and was not on any anti-coagulants. The patient was a non-smoker and denied alcohol intake.

Physical examination revealed an overweight male with a soft, non-tender but distended abdomen. He had a gross irreducible left-sided inguinoscrotal swelling which was mildly tender on palpation. There was no bruising or overlying skin changes. Examination of the right groin was unremarkable. His baseline observations were entirely normal with haemodynamic stability. Urinalysis did not show any evidence of infection.

The patient was diagnosed clinically with an irreducible large left inguinoscrotal hernia and counselled for surgery.

An open approach was adopted, with an incision made in the groin crease. The regional anatomy proved difficult to identify, and no obvious hernial sac could be found. The decision was made to convert to a laparoscopic totally extraperitoneal (TEP) approach. A large collection of old blood and clots was found in the extraperitoneal plane tracking down to the scrotum (Video 1). This was subsequently evacuated with copious washout. A thorough inspection of the region did not reveal an obvious source for the bleeding but spontaneous rupture of inferior epigastric vessels was suspected. Intrabdominal laparoscopy did not reveal any further abnormalities, and no obvious hernia was identified. Two Redivac drains were left in the cavity post-procedure, and the patient was admitted overnight for observation. The following morning his urinary catheter was removed and he was deemed fit for discharge.

**Video 1:** Laparoscopic TEP view demonstrating a large cavity containing old and altered blood. The source of the bleeding is suspected to have arisen from the inferior epigastric vessels. TEP: totally extraperitoneal

The patient was followed up 7 days post-operatively in clinic and both Redivac drains with minimal haemoserous fluid were removed. A further review of the patient several weeks later found that he had recovered well with no signs of wound infection.

## Discussion

Differential diagnoses of groin swelling include inguinal hernia, abscesses, haematomas, aneurysms, tumours, and lymphadenopathy. Additionally, in males, pathology arising from the testes, cord, and scrotal structures must also be considered.

Careful history taking, physical examination, and the use of imaging modalities where appropriate can help to distinguish between these and guide further management. Imaging is seldom used in the diagnosis of inguinoscrotal hernias, especially in cases of high clinical suspicion, but may be employed in diagnostic uncertainty. The preferred mode of investigation is a computed tomography (CT) scan with contrast, especially in hernias likely to contain strangulated/incarcerated bowel. Alternatively, ultrasound scanning is a sensitive, specific, and contrast-free mode of imaging, but its quality remains operator-dependent.

Inguinoscrotal hernias have a prevalence of 4% in males over the age of 45 years in the United Kingdom [9]. Patients often present with a fluctuating groin swelling and mild discomfort [9]. Symptoms can develop over a long period of time, with many hernias remaining asymptomatic. Complications can arise including incarceration or strangulation of the hernia, often requiring emergent intervention. Symptomatic hernias are repaired through either an open or laparoscopic approach with the defect re-enforced with mesh or sutures.

By contrast, spontaneous haemorrhage secondary to ruptured inferior epigastric vessels leading to groin swelling and pain is a rarely reported presentation [5-8]. It most commonly presents as a central abdominal wall swelling with features of shock [5,8] often necessitating urgent surgical exploration and haemostasis [5,6,8]. Increasingly, interventional radiology procedures to control bleeding may also be considered in more stable patients. Patients without any adverse features or signs of haemodynamic instability may be managed conservatively [7,8].

In this case report, imaging was precluded due to the clinical suspicion of inguinoscrotal hernia. The patient’s occupation in manual labour together with extended history, haemodynamic stability, the lack of an obvious trigger for vessel rupture, and the examination findings made a haemorrhagic cause for his symptoms unlikely. The unusual peri-operative findings coupled with this presentation are indeed a rare finding.

## Conclusions

Although the differential diagnoses of groin swellings are numerous, consideration should be given to the more uncommon presentations such as haematoma. Certainly, a history of localised abdominal trauma or the presence of shock should make clinicians suspicious of a haemorrhagic process. In cases where there is diagnostic uncertainty, imaging modalities can be utilised to aid assessment and help in further management.

Management of such patients is usually dictated by the clinical presentation. In cases where there is haemodynamic stability, conservative management can be considered. Increasingly, interventional radiology offers an additional route of management with the option of embolisation. Ultimately, in cases where there is unstable haemorrhage, surgery with ligation of the offending vessels remains a viable option.
